# Impact of *MASP2* gene polymorphism and gene-tea drinking interaction on susceptibility to tuberculosis

**DOI:** 10.1038/s41598-021-86129-x

**Published:** 2021-03-22

**Authors:** Zihao Li, Mian Wang, Hua Zhong, Xin Huang, Xinyin Wu, Xian Zhang, Jing Wang, Jing Deng, Mengshi Chen, Lizhang Chen, Hongzhuan Tan

**Affiliations:** 1 Department of Epidemiology and Health Statistics, Xiangya School of Public Health, Central South University, 110 Xiangya Road, Changsha, 410000 China; 2 Department of Cardiology, Xiangya Hospital, Central South University, Changsha, China; 3 Department of Epidemiology and Health Statistics, Hunan Normal University, Changsha, China; 4 Hunan Provincial Key Laboratory of Clinical Epidemiology, Central South University, Changsha, China

**Keywords:** Genetic association study, Risk factors

## Abstract

Mannan-binding lectin-associated serine protease-2 (MASP-2) has been reported to play an important role as a key enzyme in the lectin pathway of the complement system. The objectives of our study were to determine whether the single-nucleotide polymorphism (SNPs) of *MASP2* and the gene-tea drinking interaction were associated with the susceptibility to TB. In total, 503 patients and 494 healthy controls were contained. Three SNPs (rs12142107, rs12711521, and rs7548659) were genotyped. The association between the SNPs and susceptibility to TB were investigated by conducting multivariate unconditional logistic regression analysis. The gene-tea drinking interactions were analyzed by the additive model of marginal structural linear odds models. Both genotype AC + AA at rs12711521 of *MASP2* genes and genotype GT + GG at rs7548659 of *MASP2* genes were more prevalent in the TB patient group than the healthy control group (OR: 1.423 and 1.439, respectively, P < 0.05). In addition, The relative excess risk of interaction (RERI) between tea drinking and rs12142107, rs12711521, and rs7548659 of *MASP2* genes was found to suggest negative interactions, which reached − 0.2311 (95% confidence interval (CI): − 0.4736, − 0.0113), − 0.7080 (95% CI − 1.3998, − 0.0163), and − 0.5140 (95% CI − 0.8988, − 0.1291), respectively (P < 0.05). Our finding indicated that the SNPs (rs12711521 and rs7548659) of *MASP2* were associated with the susceptibility to TB. Furthermore, there were negative interactions between tea drinking and rs12142107, rs12711521, and rs75548659 of *MASP2* gene, respectively. Our research provides a basis for studying the pathogenesis and prevention of tuberculosis.

## Introduction

Tuberculosis (TB), an airborne infection disease caused by *Mycobacterium tuberculosis* (MTB), is one of the top 10 causes of death worldwide and the leading cause of death from a single infectious agent (ranking above human immunodeficiency virus/acquired immunodeficiency syndrome (HIV/AIDS)). According to the 2020 World Health Organization (WHO) report, there were an estimated 10 million new cases of TB worldwide and approximately 1.5 million deaths among HIV-negative people in 2018. China has the second highest TB burden in the world, accounting for 9% of the world’s TB cases^1^.

The MTB can be non-specifically cleared early in the infection via the innate immune system before the 2–3 week delay of adaptive immunity^2^. The complement system is one of the major effectors of the innate immunity and an important bridge between innate and adaptive immunity^3^. It mediates many cellular and humoral interactions within the immune response, including chemotaxis, phagocytosis, cell adhesion, B-cell differentiation and T-cell response^4^. As a fast and effective immune surveillance system, complement has a significant impact on healthy and altered host cells and foreign invaders^5^. Three major pathways activate the complement system, which are known as the classical, alternative, and lectin pathways^6^.

The complement lectin pathway is activated by the binding of pattern recognition molecules such as mannose bind lectin (MBL), ficolins (H-ficolin, L-ficolin, or M-ficolin) or collectin (kidney 1, CL-K1 and collectin liver 1, CL-L1). Mannan-binding lectin-associated serine protease-2 (MASP-2) is activated by the combination of these pattern recognition molecules and triggers the downstream complement to initiate a series of cascades^7^.

The *MASP2* gene is located on the chromosome 1p36.23–31 and encodes two proteins, MASP-2 and Map19^8^. Both the levels and function of MASP-2 are modulated by the single-nucleotide polymorphisms (SNPs) of the *MASP2* gene^9,10^. The level of MASP-2 in plasma plays a dual role in the infection and progression of infectious diseases^11,12^. The deficiency of MASP-2 in plasma has been associated with the susceptibility of HIV. By contrast, the overproduction of MASP-2 may increase the inflammatory effect. In other words, the low levels of MASP-2 have protective effect in the progression of HIV^12^. Moreover, in recent years, there have been a large number of previous studies proving the association between the polymorphism of *MASP2* gene and the susceptibility of infective diseases and disease of immune system^13–16^. However, few articles have investigated the association between *MASP2* gene polymorphism and susceptibility to TB. A result of a meta-analysis showed that *MASP2* gene (rs72550870) polymorphism was not associated with infectious diseases^17^. While a recent study reported three instances of *MASP2* rs72550870 variant, two in people with pulmonary TB and one completely healthy person. All the three subjects had low MASP-2 concentrations and low MBL-MASP-2 complex activities^18^. However, whether *MASP2* mutations increase predisposition to TB remains to be confirmed in a wider population. In the Chinese Han population, two previous studies found that the SNPs rs2273346 and rs6695096 of *MASP2* genes can increase susceptibility to TB^19,20^. Therefore, we speculate that other SNPs of the *MASP2* gene may confer predisposition or protection from TB. So, we designed a case–control study to investigate the association between rs12142107 (on the exon 8), rs12711521 (on the exon 12), and rs7548659 (on the promotor) of *MASP2* genes and susceptibility of TB.

Tea, which is made from the soaking of the leaves of *Camellia sinensis*, is a very popular common beverage all over the world, especially in China^21^. Recent epidemiologic evidence has shown that tea drinking also has a certain protective effect on the occurrence and development of TB^22,23^. Epigallocatechin gallate, the main bioactive ingredient of tea, showed the best activity and selectivity as a *Mycobacterium tuberculosis* (Mtb) dihydrofolate reductase inhibitor^24^. Several experimental investigations have been conducted on this phenomenon, one of which is that epigallocatechin-3-gallate (EGCG) in the tea leaves can downregulate the expression of the host molecule tryptophan-aspartate containing coat protein (TACO) gene and inhibit MTB survival within macrophages^25^. The complement system plays an important role in innate and adaptive immunity, including opsonization and phagocytosis^26^. As an important factor of the complement lectin pathway, MASP-2 may resist the invasion of MTB through opsonization and phagocytosis. Therefore, EGCG in tea leaves and MASP-2 can both interact with macrophages directly or indirectly to affect the host infection with MTB. Therefore, our research also explored *MASP2* gene-tea drinking interactions on the TB susceptibility to provide a better basis for further research on the role of *MASP2* polymorphisms and tea drinking on TB.

## Methods

### Source of subject

In this case–control study, we adopted stratified sampling to acquire the sample. Firstly, we selected four county-level Centers for Disease Control (CDCs) (i.e., Qidong County CDC, Yueyanglou District CDC, Yueyang County CDC, and hongjiang City CDC) by using a random number table among 122 counties/cities/districts in Hunan Province, China. Next, we randomly selected cases from all TB patients newly registered. All cases were TB patients based on the diagnostic criteria for TB^27^. We also used stratified sampling strategy to select healthy controls. Firstly, we used a random number table containing 14 community health service centers to select a health service center (i.e., xingang Community Health Service Center) in Kaifu District, Changsha City. Next, one of the six communities (i.e., Xin’ansi Community) covered by the Xingang Community Health Service Center was selected randomly. Selection criteria for controls: (1). with a bacille Calmette–Guerin (BCG) scar and average diameter of PPD (purified protein derivative (PPD) 10 mm, or without a BCG scar and a history of BCG vaccination, but the average diameter of PPD induration was 5 mm; (2) no abnormalities on chest X-ray. All subjects were unrelated ethnic Han Chinese. Exclusion criteria for both groups were human immunodeficiency virus (HIV) infection, diabetes mellitus, cancer, organ transplantation, autoimmune disease, long-term use of hormones, primary immunodeficiency, and treatment with immunosuppressive drugs.

All protocols were approved by the Xiangya School of Public Health Central South University Ethics Review Committee (XYGW-2018-11). Written informed consent to participate in this study was obtained from each study participant. A detailed questionnaire was designed to collect information of demographic characteristics and selected information, which included sex, age, marital status, educational background, body mass index (BMI), smoking status, alcohol drinking, and tea drinking. The tea drinkers are defined as the people who drink tea one cup a day at least, last for over half one year. Five milliliters of venous blood from each participant was aseptically collected in ethylenediaminetetraacetic acid (EDTA) anticoagulant tubes and stored in a 4 °C refrigerator before use. A blood DNA kit provided by Shanghai Sangon Biotech Co., Ltd. was then used to extract the peripheral white blood cell genome.

### Size of sample

According to the sample size calculation formula designed for 1:1 unmatched case team study, we estimated that the sample size required for each case group and control group was 442 cases (estimated Minor Allele Frequency (MAF) was 0.13, estimated odds ratio (OR) was 1.8, α = 0.05, β = 0.1).

### Selection basis of SNPs of *MASP2* gene

In this study, the basis for the selection of SNPs of the *MASP2* gene mainly includes the following two points: Firstly, we selected the sites, which were reportedly associated with TB susceptibility on PubMed; secondly, the sites we selected were polymorphic sites associated with other infectious diseases reported in the literature. To ensure the statistical efficacy in this study, the minimum allele frequency (MAF) of the selected polymorphic site was required to be ≥ 0.05. Based on the relevant gene loci data published in the database, we learned the frequencies of corresponding SNPs of *MASP2* gene by searching for corresponding SNPs on the dbSNP database (http://www.ncbi.nlm.nih.gov). Finally, we chose the rs12142107, rs12711521, and rs7548659 of *MASP2* gene.

### Genotyping

A Wizard Genomic DNA Purification Kit (Promega) was used for peripheral white blood cell genome extraction, and the quality-controlled DNA was preserved at − 20 °C until assay. The site sequences of rs12141207, rs7548659, and rs12711521 of the *MASP2* gene were identified in the Gene bank, and Assay Design 3.1 (Sequenom) was used to design appropriate primers. The synthesized primers were inspected for quality inspection by matrix-assisted laser desorption ionization time-of-flight mass spectrometry (MALDI-TOF). The polymerase chain reaction (PCR) reaction system was 5 μl, including 1.8 μl of ddH2O, 1 μl of PCR primer mix, 1 μl of gDNA (20–50 ng), 0.5 μl of 10*PCR buffer, 0.4 μl of MgCl2 (25 mM), 0.2 μl of Hotstar, and 0.1 μl of dNTP (25 mM). The condition of reaction was 95 °C predegeneration for 2 min, amplification (95 °C for 30 s, 56 °C for 30 s, 72 °C for 60 s) for 45 cycles, and extension at 72 °C for 5 min. The enzyme digestion reaction system was 2 μl, including 1.53 μl of ddH_2_O, 0.17 μl of SAP buffer, and 0.3 μl of SAP enzyme. The reaction condition was 37 °C for 40 min and 85 °C for 5 min. The single base extension reaction system was 2 μl, including 0.94 μl of extend primer mix, 0.619 μl of ddH_2_O, 0.041 μl of iplex enzyme, 0.2 μl of iplex buffer, and 0.2 μl of terminator mix. The corresponding reaction condition was 94 °C predegeneration for 30 s, 40 cycles of amplification (5 cycles of three temperature settings: 94 °C for 5 s, 80 °C for 5 s, 52 °C for 5 s, and 72 °C extension for 3 min). Subsequently, we plated the clean resin into a 6-mg resin plate to purify the resin, and the resin-extended extension product was transferred to a 384-well SPECTROCHIP (SEQUENOM) chip for spotting (MASSARRAY NANODISPENSER RS1000). SEQUENOM MASSARRAY SNP assay could reflect the base differences caused by SNP polymorphism into molecular weight differences. MALDI-TOF was used to detect the molecular weight of the extension product, and the analysis was performed using MASSARRAY TYPER 4.0. SNP typing could be determined by the difference in molecular weight.

### Statistical analysis

Epidata 3.0 was used to input data, and SAS 9.2 was used to analyze the data. Categorical variables were presented as proportions. Chi-squared (χ^2^) test was conducted for the comparison of grouped data, and P < 0.05 was considered significant. Hardy–Weinberg Equilibrium (HWE) was tested using chi-square (χ^2^) tests for each SNP. The risk associated with individual alleles was calculated as the odds ratio with 95% confidence interval (CI). To exclude possible confounding risk factors, the occurrence of TB was used as the dependent variable, the rs12142107, rs12711521, and rs7548659 of *MASP2* genes were used as the independent variables, and the sex, age, marital status, educational background, BMI, smoking status, alcohol drinking, and tea drinking were used as the covariates, and multivariate unconditional logistic regression analysis conducted. The additive interaction of marginal structural linear odds models were used for point estimation and interval estimation of the relative excess risk of interaction (RERI) to analyze the gene-environment interaction^28^. RERI > 0 suggests positive interactions.

### Ethics declarations

All protocols were approved by the Xiangya School of Public Health Central South University Ethics Review Committee (XYGW-2018-11). all experiments were performed in accordance with relevant named guidelines and regulations. Informed consent was obtained from all participants.

## Results

503 TB patients and 494 healthy controls were included in this study. We investigated the association of three *MASP2* SNPs and TB susceptibility using a case–control study. There was a statistical difference in BMI, history of BCG vaccination, smoking and tea drinking between the case group and the control group (P < 0.05), but no statistical difference was found in gender, age, marital status, educational background and alcohol consumption (P > 0.05), the data was cited from reference^28^.

The genotypic frequencies of *MASP2* polymorphism in TB patients and healthy controls are shown in the Table 1. Multivariate unconditional logistic regression analysis showed that rs12711521 and rs7548659 of *MASP2* genes were associated with susceptibility to TB. Genotype AC and CC at rs12711521 (compared with genotype AA) and genotype GT and GG at rs7548659 (compared with genotype TT) were found to have significant association with increased risk of TB (OR = 1.323, 1.869, 1.350, and 2.196, respectively, P < 0.05). However, the mutation at rs12142107 was not associated with susceptibility to TB. The dominant model of rs12711521 (AC + CC vs. AA) and rs7548659 (GT + GG vs. TT) were also found to have statistically significant association with increased risk of TB (OR reaching 1.423 and 1.439, P < 0.05).

**Table 1:** *MASP2* gene polymorphism versus TB incidence.

	TB patients (n = 503)		Controls (n = 494)		χ^2^	*P* value	cOR (95%CI)	aOR (95%CI)^a^
	n	%	n	%				
**rs12142107**								
CC	393	78.13	402	81.38	2.206	0.332	1	1
CT	101	20.08	87	17.61			1.188 (0.863–1.633)	1.167 (0.840–1.623)
TT	9	1.79	5	1.01			1.841 (0.612–5.543)	1.465 (0.474–4.535)
CT + TT	110	21.87	92	18.62			1.223 (0.897–1.667)	1.185 (0.860–1.633)
**rs12711521**								
AA	192	38.17	242	48.99	14.748	0.001	1	1
AC	233	46.32	204	41.3			1.440 (1.102–1.880)*	1.323 (1.004–1.743)*
CC	78	15.51	48	9.72			2.048 (1.364–3.075)*	1.869 (1.227–2.847)*
AC + CC	311	61.83	252	50.75			1.556 (1.209–2.001)*	1.423 (1.095–1.849)*
**rs7548659**								
TT	239	47.51	286	57.89	14.712	0.001	1	1
GT	219	43.54	186	37.65			1.409 (1.086–1.828)*	1.350 (1.029–1.772)*
GG	45	8.95	22	4.45			2.448 (1.429–4.192)*	2.196 (1.263–3.817)*
GT + GG	264	52.49	208	42.11			1.519 (1.183–1.950)*	1.439 (1.106–1.871)*

For Hardy–Weinberg equilibrium detection, P > 0.05.

*P < 0.05.

^a^A multivariate logistic regression model was used to adjust the covariates of sex, age, marital status, educational background, BMI, smoking status, alcohol drinking, tea drinking, and BCG vaccination.

The additive model of marginal structural linear odds models was used to analyze the impact of the interactions between *MASP2* genes and tea drinking on susceptibility to TB. We adjusted for the covariates of sex, age, marital status, educational background, BMI, smoking status, alcohol drinking, and history of BCG vaccination. The RERI between tea drinking and rs12142107, rs12711521, and rs7548659 of *MASP2* genes was found to be − 0.2311 (95% CI − 0.4736, − 0.0113), − 0.7080 (95% CI − 1.3998, − 0.0163), and − 0.5140 (95% CI − 0.8988, − 0.1291), respectively (P < 0.05), which suggests negative interactions (Table 2).

**Table 2:** Impact of interactions between *MASP2* SNPs and tea drinking on the incidence of TB.

	Tea drinking		cRERI	aRERI (95% CI)^a^
	No	Yes		
**rs12142107**				
CC	1	0.665	− 0.1533	− 0.2311 (− 0.4736, − 0.0113)*
CT + TT	1.298	0.81		
**rs12711521**				
AA	1	0.664		− 0.7080 (− 1.3998, − 0.0163)*
AC + CC	1.509	1.028	− 0.1459	
**rs7548659**				
TT	1	0.757		− 0.5140 (− 0.8988, − 0.1291)*
GT + GG	1.743	1.01	− 0.4908	

*p < 0.05.

^a^A multivariate logistic regression model was used to adjust for the covariates of sex, age, marital status, educational background, BMI, alcohol drinking, smoking, and BCG vaccination.

## Discussion

Our study aimed to evaluate the relationship between three *MASP2* SNPs and TB. Our data showed that the AC + AA genotype of rs12711521 and the GT + GG genotype of rs7548659 was more prevalent in TB patients than healthy controls, which demonstrated that the rs12711521 and rs7548659 of *MASP2* gene were related to the increased susceptibility of TB, while no such association was found in the rs12142107 of *MASP2* gene.

SNP rs12711521, located in the exon10 region of *MASP2*, was found to be associated with susceptibility to HTLV-1 infection in a study in northwestern Brazil (OR = 2.71 [95%CI = 1.56–4.74], P < 0.001)^29^. Another study involving 104 South-Brazilian patients with chronic hepatitis C found an association of rs12711521 variant with susceptibility to HCV (OR = 6.33 [95%CI = 1.85–21.7], P = 0.003. Previous studies found amino acid changes at the position rs12711521 would cause the cleavage region of the catalytic enzyme and change the substrate specificity^30^. In this study, we speculated the rs12711521 located in the exon10 could lead to the change of aspartic acid 371 to tyrosine and might influence secondary interaction with C4b and activation of complement system^12^. Our study first found the association between rs7548659 of the *MASP2* gene and susceptibility to TB. Although the rs7548659 is located in the promoter region of *MASP2*, as a central component in the initiation of transcription, the promoter mutation may influence the regulation of gene expression^34^, which subsequently results in the decrease of *MASP2* level and activation of complement. However, our study found that the rs12142107 of *MASP2* had no association with the predisposition to TB, possibly because the mutation at the rs12142107 site is a synonymous mutation (Ala > Ala) and does not affect gene expression^34^.

Our findings suggest that the polymorphism of *MASP2* gene may increase the susceptibility to TB. This is consistent with the results of two previous studies^20,21^. This result was also supported in two recent animal experiments. Two studies applied human-MASP-2 to mouse and rabbit models, respectively, and found protective effects of MASP-2 on TB infection^31,32^. Not only that, it has also been found in humans that mutations in certain sites of the *MASP2* gene increase the susceptibility to TB. Sokolowska et al.^18^ found in two Polish pulmonary TB patients and a healthy control that they all had a decrease in MASP-2 concentration and a reduction in MBL-MASP-2 activity, with MASP-2 concentration levels lower than the fifth percentile of Polish healthy adults. Moreover, the healthy control is a G/G homozygote of the SNP rs72550870 mutation. This phenomenon suggests that the mutation of *MASP2* may lead to decrease of the MASP-2 serum level. MASP-2 can cleave C4 and C2, which are key to activate downstream complement components. Subsequently, C3 and C5 were cleaved to form the membrane attack complex and produced soluble and surface bound cleavage products, including C4a, C2a, C3a, C3b and C5a, which regulate inflammation serving as chemical attractants, and activators of innate immune cells (such as lymphocytes and macrophages)^32^. Thereby, the polymorphism of *MASP2* (rs12711521 and rs7548659) reduced the body's defense ability against pathogens and increased the susceptibility to TB.

However, several studies have also found that MASP-2 has no effect on susceptibility to TB and even promotes the development of TB. In a BCG-infected mice model experiment, Gao et al.^33^ found that TB granulomas of mice in the human MASP-2 CCP1/2SP nanolipoplexes-treated group had enlarged and that the bacterial load had not decreased, which indicated that MASP-2 may promote the inflammatory response caused by TB infection. Similarly, Boldt et al.^34^ also found that elevated MASP-2 levels might be of prognostic value for leprosy progression. Chalmers et al.^35^ found that there was no difference in MASP-2 serum levels or genotype between TB patients and controls in the Indian population. However, they speculated that the sample size was not powered to detect small differences in genotype frequencies between TB patients and controls.

In our study, marginal structural linear odds model analysis showed that there were negative interactions between rs12142107, rs12711521 and rs7548659 of *MASP2* gene and tea drinking. To better control the occurrence of TB in real life, people with the above mutations should be encouraged to cultivate a habit of tea drinking, which has some effect on controlling the TB infection. Tea, which is made from the soaking of the leaves of *Camellia sinensis*, is the most common beverage in the world except water^36^. Some epidemiological evidences suggest that tea drinking habits may have some protective effect on the TB infection, and there was a dose-dependent relationship between them^22,23^. In addition, results of a randomized controlled trial demonstrated that green tea extract (GTE) decreases the risk of delay in sputum smear conversion^37^. A recent study found a mixed flavonoid supplement containing green tea extract have a strong effect against M. tuberculosis infectivity in macrophages by the enrichment of glutathione and regulation of cytokine^38^. Our research found a negative interaction between *MASP2* gene polymorphism (rs12142107, rs12711521 and rs7548659) and tea drinking on TB susceptibility, which might provide a new idea to study the preventive effect of tea on TB. Previous research suggested that the synergistic effect of high MBL levels and MBL-associated serine protease (MASP) might mediate complement activation and uptake of M. tuberculosis by complement receptors on the macrophage^39^. Therefore, the GTE and MASP-2 can both interact with macrophages directly or indirectly to affect the MTB infection within host. Our study also found *MASP2* gene polymorphism (rs12711521 and rs75548659) conferred susceptibility to TB, which was helpful to understand the pathology of TB, identify the risk factors of TB, and take targeted population prevention measures.

There were limitations in our study. On the one hand, we only determined three SNPs of *MASP2* genes, but the changes of serum MASP-2 and MAp19 level due to mutations need further studies. Therefore, it is necessary to design a study to further investigate the relationship between serum MASP-2 and MAp19 level and TB susceptibility. On the other hand, the molecular mechanism of how *MASP2* gene polymorphism affects the occurrence and development of TB needs more exploration in future studies. Despite these limitations, we minimized the possibility of bias through the entire process, including sample selection, data selection, and statistical analysis. In any case, our results are reliable, which is of great significance for identifying people at high risk for TB as well as the prevention of TB.

## Conclusion

We suggest that *MASP2* gene polymorphism (rs12711521 and rs75548659) confers susceptibility to TB, and there were negative interactions between tea drinking and rs12142107, rs12711521, and rs75548659 of *MASP2*, respectively. Finally, the molecular mechanism of how *MASP2* gene polymorphism and the gene-tea drinking interaction affects the occurrence and development of TB needs more exploration.
